# Non-alcoholic fatty liver disease in Tanzania: prevalence, determinants, and diagnostic performance of triglycerides-glucose index and triglycerides-glucose index –body mass index compared to the hepatic ultrasound in overweight and obese individuals

**DOI:** 10.1186/s12876-024-03164-4

**Published:** 2024-03-04

**Authors:** Semvua B. Kilonzo, Eliud Kamala, Hyasinta Jaka, Patrick Ngoya

**Affiliations:** 1 Department of Internal Medicine, Catholic University of Health and Allied Sciences, P.O. Box 1464, Mwanza, Tanzania; 2 Department of Internal Medicine, Bugando Medical Centre, P.O. Box 1370, Mwanza, Tanzania; 3 Department of Radiology, Catholic University of Health and Allied Sciences, P.O. Box 1464, Mwanza, Tanzania

**Keywords:** Fatty, Liver, TyG, Overweight, Obesity, Tanzania

## Abstract

**Background:**

Non-alcoholic fatty liver disease (NAFLD), which is closely associated with metabolic syndrome (MetS), is rarely reported in Tanzania, where MetS is prevalent. The purpose of this study was to determine the prevalence and associated factors of this condition in overweight and obese individuals and to correlate standard ultrasound diagnosis with triglyceride-glucose index (TyG) and TyG-body mass index (TyG-BMI).

**Methods:**

A cross-sectional analysis was performed in 181 adult outpatients attending a general medical clinic. The presence of fatty liver was detected by ultrasound. Demographic, clinical, and laboratory data were collected and analyzed using STATA 15. To compare categorical variables, a chi-square test was employed, while a Student’s t-test was used to compare continuous variables. Additionally, a multivariate regression analysis was conducted to identify the determinants of NAFLD. A significance level was set at *p* < 0.05. The discriminatory power of TyG and TyG-BMI for diagnosing NAFLD was evaluated using Receiver Operating Characteristic (ROC) Curve analysis and the Area Under the ROC Curve (AUC) was reported.

**Results:**

The overall prevalence of NAFLD was 30.4% (55/181). The prevalence’s of NAFLD in patients with class III obesity, class II obesity, class I obesity and overweight were 50.0% (12/24),, 38% (19/50), 23.7% (18/76), and 19.5% (6/31),respectively. NAFLD was strongly predicted by hyperuricemia (≥ 360 μmol/L) (*p* = 0.04) and TyG ≥ 8.99 (*p* = 0.003). The best cut-off values of TyG and TyG-BMI to predict NAFLD were 8.99 [AUC 0.735; sensitivity 70.9%, specificity 79.3%] and 312 [AUC 0.711; sensitivity 60% and specificity 75.4%] respectively.

**Conclusions:**

The prevalence of NAFLD is high among people with overweight and obesity in Tanzania. We did not find sufficient evidence to recommend the use of TyG and TyG-BMI as surrogates for hepatic ultrasound in detecting NAFLD, and further evaluation is recommended.

## Background

Non-alcoholic fatty liver disease (NAFLD) refers to the presence of hepatic steatosis that affects more than 5% of hepatocytes without secondary causes of hepatic fat accumulation, such as excessive alcohol consumption, long-term use of steatogenic drugs, and any other liver diseases. NAFLD can present as simple steatosis without hepatocyte damage or a more severe form, non-alcoholic steatohepatitis (NASH), in which there is inflammation and damage of the hepatocytes (ballooning) with or without liver fibrosis. NASH has been associated with an increased risk of progression to cirrhosis and / or hepatocellular carcinoma (HCC) and other metabolic conditions such as obesity and type 2 diabetes mellitus (DM) [1]. NAFLD has also been associated with increased mortality compared to the general population [2].

The prevalence of NAFLD is estimated to be around 32% in the general population worldwide, which has increased significantly over time compared to previously reported of around 25% in studies conducted before 2006 [3]. The prevalence of NAFLD in Africa is often underestimated and underreported due to scarcity of comprehensive data. Studies that are available indicate an average prevalence of 14% in the region [4]. Furthermore, the occurrence of NAFLD is associated with a high burden of underlying metabolic comorbidities. A recent meta-analysis reported that in patients with NAFLD, dyslipidemia, obesity, metabolic syndrome (MetS), and DM occurred in 69, 51, 41, and 23%, respectively [4]. Due to the growing burden of DM (10.3%), obesity (32.4%) and MetS (38%) in Tanzania [5–7], there may be many cases of NAFLD that go unreported. To our knowledge, there is no published study showing the spectrum of NAFLD in the country.

Abdominal ultrasound is an effective diagnostic test for liver steatosis and is highly recommended by several guidelines [1, 8, 9]. According to these guidelines, liver biopsies are generally reserved for those whose results of ultrasound or other non-invasive tests are inconclusive and those who are at high risk of progressing to advanced fibrosis due to its invasive nature and associated costs. Although feasible in low-resource environments, ultrasound is an operator-dependent procedure that requires highly trained personnel. It has also been observed that its sensitivity is significantly reduced in the presence of mild steatosis and in morbidly obese subjects [10]. Therefore, there is still a need for simpler and more suitable tests to diagnose hepatic steatosis. Given the close relationship that exists between NAFLD and MetS, the occurrence of MetS features; triglyceride, insulin resistance, and obesity also correlate with the presence of NAFLD. Recent meta-analyses have found a strong prediction of NAFLD using the triglyceride glucose index (TyG) and the TyG-body mass index (TyG-BMI) [11, 12]. Data on the use of TyG and TyG-BMI to predict liver steatosis are scarce in Africa.

Therefore, this study aims to determine the prevalence of NAFLD and its determinants in overweight and obese individuals in an outpatient setting in Tanzania. Also, this study aims to assess the diagnostic performance of TyG and TyG-BMI compared to the hepatic ultrasound.

## Methods

### Study design, population, and setting

This was a cross-sectional study conducted on 181 patients between 28 February and 03 May 2022 at the medical outpatient clinic of the Bugando Medical Center (BMC) in northwest Tanzania. BMC is a tertiary and teaching hospital in the Lake Zone of Tanzania, serving approximately 13 million people. The hospital provides inpatient and outpatient services with an approximate bed capacity of 1000.

Participants were the patients who attended the clinic for various medical conditions aged 18 years or older, had a body mass index (BMI) ≥ 25 kg / m^2^ and consented to the study. Patients were excluded if they had a previous diagnosis of liver disease or had a history of known secondary causes of hepatic steatosis that included autoimmune or viral hepatitis, Wilson’s disease, hemochromatosis, and excessive alcohol consumption.

### Sample size determination

The sample size was calculated using the Kish and Leslie formula as stated elsewhere [7].

$$\frac{{\textrm{Z}}^2\textrm{PQ}}{{\textrm{d}}^2}$$

Where, Z = 1.96 (95% CI), *P* = 0.12 (12% prevalence of NAFLD in previous study) [4] Q = 1-P and d = margin of error, that is 0.05.

The minimum sample size was 162, and we recruited 181 participants.

### Data collection

Using a standardized questionnaire, demographic information such as age, sex, occupation, place of residence, marital status, and level of education was collected from clinic records and personal interviews. A detailed history of alcohol consumption was also obtained from patients and excessive alcohol consumption was described as follows; more than one drink per day for women and more than two drinks per day for men. An alcoholic drink is equivalent to 14 g of pure alcohol [1]. A medical history was also taken that focused on the existence of diabetes mellitus, hypertension, malignancies, and a significant use of medications that have the potential to cause fatty liver, such as corticosteroids, valporic acid, methotrexate, tamoxifen, and amiodarone.

Each subject was subjected to a physical examination, which included measurement of blood pressure, height, weight, and waist circumference according to the available guidelines [13]. In brief, a trained nurse utilized an OMRON HEM 7130 blood pressure monitor (OMRON Healthcare, Kyoto, Japan) to measure the participants’ blood pressure (BPs). Prior to the measurement, participants were instructed to rest unattended for 5 minutes, sitting with their back supported, legs uncrossed, and feet flat on the floor. Three consecutive BP measurements were then taken on the right arm, with a 60-second interval between each measurement. The average of all three measurements was used for further analysis.

Weight was measured using a Seca® 813 digital weight measuring scale in kilograms (kg) (Seca, Hamburg, Germany). Participants were instructed to wear minimal clothing and remove their shoes during the measurement. Height was measured with a Seca® 213 stadiometer in centimeters (cm). Waist and hip circumferences were measured twice using a 203 cm Seca® measuring tape, The average of the two measurements was used for each of these recorded values.

Body mass index (BMI) was estimated using the formula:

$$\textrm{BMI}\kern0.75em =\kern1em \frac{\textrm{Weight}\ \left(\textrm{kg}\right)}{\textrm{Height}\ \left({\textrm{cm}}^2\right)\ }$$

BMI was classified as overweight (BMI 25–29.9 kg / m^2^), obesity class I (BMI 30–34.9 kg / m^2^), obesity class II (BMI 35–39.9 kg / m^2^), and obesity class III (BMI ≥ 40 kg / m^2^) [6].

Approximately 15 ml of blood samples were taken from each participant after fasting overnight (> 8 hours) for measurements of fasting blood glucose (FBG), fasting serum triglyceride (TG), serum high-density lipoproteins (HDL) and uric acid. Blood chemistry measurements were analyzed using a COBAS 400 PLUS automated chemistry machine. The TyG of each participant was estimated using the formula [12]:

$$\textrm{TyG}=\textrm{Ln}\ \Big[\textrm{fastig}\ \textrm{triglycerides}\ \left(\textrm{mg}/\textrm{dl}\right)\textrm{x}\kern0.5em \textrm{fasting}\ \textrm{blood}\ \textrm{glucose}\ \left(\textrm{mg}/\textrm{dl}\right)$$

TyG-BMI was estimated using the formula [11]:

$$\textrm{TyG}-\textrm{BMI}=\textrm{TyG}\ \textrm{x}\kern0.5em \textrm{BMI}\ \left(\textrm{kg}/{\textrm{m}}^2\right)$$

Abdominal ultrasound was performed by an experienced radiologist to ensure inter-observer consistency, using the ALPINION E-Cube 8 LO 1380 device at 3.5 Hz to determine the presence of hepatic steatosis. Fatty liver was diagnosed if there was increased hepatic echo as previously described [14].

### Statistical analyses

Data entry was performed using Microsoft Excel before being exported to STATA Version 15 (San Antonio, Texas) for analysis. In this study, categorical variables were expressed in frequencies and ratios and compared using the chi-square test. Continuous variables were expressed as medians with interquartile ranges (IQR) and compared using Student’s t test. When determining the predictors of NAFLD, the variables with *p* < 0.1 were integrated in the multiple regression analysis. The level of significance was established at *p* < 0.05. The receiver operating characteristic (ROC) curve analysis was used to determine the optimal cut-off points of TyG and TyG-BMI for the diagnosis of NAFLD. Sensitivity, specificity, positive predictive value (PPV) and negative predictive value (NPV) were calculated and reported.

## Results

During the study period, 763 patients were examined for eligibility, of which 302 were eligible for recruitment. Of these, 121 participants were excluded due to incomplete investigations, leaving 181 patients for analysis.

### Characteristics of the study participants

We recruited a total of 181 overweight and obese patients attending the general medical outpatient clinic from 28 February to 03 May 2022. Of these, 55 (30.4%) (95%CI; 24.1–37.5) were diagnosed with NAFLD. People with NAFLD were predominantly females 67.3% (37/55) versus males 32.7% (18/55), but the difference was not statistically significant (*p* = 0.57). The age group of 61 to 70 years comprised most of the population 32.0% (58/181), while younger individuals (< 30 years) comprised the minority (0.5% (1/181). Most (150/181; 82.9%) of the study participants were obese, and patients with obesity class III had significantly higher frequency of NAFLD (21.8%; 12/55) compared to those without NAFLD (9.5%; 12/126) (*p* = 0.03). Almost two-thirds (72.4%; 131/181) of all participants were hypertensive. There was a higher rate of NAFLD among patients with DM compared to those without NAFLD, but the difference was not statistically significant (54.5% vs. 39.7%, *p* = 0.06), In terms of biochemistry, the median levels of TG and uric acid were significantly higher among people with NAFLD compared to those without NAFLD (*p* < 0.05 for all). Furthermore, the median TyG [9.2 (8.8–9.5) vs 8.6 (8.4–8.9), *p* < 0.001] and TyG-BMI [320.2 (289.1–354.6) vs 286.4 (266.1–312.3), *p* < 0.001] were significantly higher among participants with NAFLD compared to those without (Table 1).

**Table 1:** Baseline characteristics of study participants in relation to the presence of Non-alcoholic fatty liver disease (*N* = 181)

Variable	Frequency (%) or median (IQR)			*P* value
	All patients (*n* = 181)	NAFLD present (*n* = 55)	NAFLD absent (*n* = 126)	
Sex				0.57
Male	54 (29.8)	18 (32.7)	36 (28.6)	
Female	127 (70.2)	37 (67.3)	90 (71.4)	
Age (years): < 30	1 (0.5)	1 (1.8)	0	α
30–40	16 (8.8)	3 (5.4)	13 (10.3)	0.29
41–50	30 (16.5)	6 (10.9)	24 (19.0)	0.18
51–60	55 (30.4)	20 (36.3)	35 (27.8)	0.25
61–70	58 (32.0)	21 (38.2)	37 (29.4)	0.24
> 70	21 (11.6)	4 (7.3)	17 (13.5)	0.23
BMI (kg/m^2^): Overweight (25–29.9)	31 (17.1)	6 (10.9)	25 (19.8)	0.14
Obesity class I (30 to 34.9)	76 (41.9)	18 (32.7)	58 (46.0)	0.09
Obesity class II (35–39.9)	50 (27.6)	19 (34.6)	31 (24.6)	0.17
Obesity class III (≥40)	24 (13.3)	12 (21.8)	12 (9.5)	0.03
Residence				0.12
Urban	93 (52.4)	33 (60.0)	60 (47.6)	
Rural	88 (48.6)	22 (40.0)	66 (52.4)	
Underlying condition				
Hypertension	131 (72.4)	44 (80.0)	87 (69.0)	0.13
Diabetes Mellitus	80 (44.2)	30 (54.5)	50 (39.7)	0.06
Hypertension and diabetes mellitus	74 (40.9)	28 (50.9)	46 (36.5)	0.07
Systolic blood pressure (mmHg)	134 (122–146)	141 (126–153)	13 3 (121–143)	0.05
Diastolic blood pressure (mmHg)	83 (73–89)	86 (74–90)	81.5 (72.0–88.0)	0.16
Waist circumference (cm)	103.5 (98–113.5)	109 (99–121)	102 (97–112)	0.006
Triglycerides (mg/dl)	1.4 (1.0–1.9)	1.9 (1.2–2.3)	1.2 (0.9–1.6)	< 0.001
High-density lipoproteins (mmol/L)	1.1 (0.9–1.4)	1.0 (0.8–1.3)	1.1 (0.9–1.4)	0.03
Fasting blood glucose (mmol/L)	5.8 (5.2–6.8)	6.4 (5.6–7.9)	5.6 (5.2–6.3)	< 0.001
Uric acid (μmol/L)	381.4 (310.6–447.8)	426 (335.7–532.0)	372.4 (295.8–423.1)	< 0.001
Triglyceride-glucose index	8.8 (8.4–9.2)	9.2 (8.8–9.5)	8.6 (8.4–8.9)	< 0.001
Triglyceride-glucose index-BMI	293.4 (268.6–324.7)	320.2 (289.1–354.6)	286.4 (266.1–312.3)	< 0.001

*IQR* Interquartile range, *NAFLD* Non-alcoholic fatty liver disease, *BMI* Body mass index

^**α**^ Not computed because of zero observation

### Prevalence of non-alcoholic fatty liver disease

The prevalence of NAFLD among people with overweight was 19.3% (6/31) and among obese people was 49/150 (32.6%). Regarding the classes of obesity, the prevalence of NAFLD was 50% (12/24) in people with class III obesity, 38.0% (19/50) in class II obesity and 23.7% (18/76) in class I obesity.

### Determinants of non-alcoholic fatty liver disease

Using multivariate logistic regression, it was found that NAFLD was strongly predicted by elevated uric acid [OR 2.1(1.1–4.7), *p* = 0.04] and TyG of 8.99 or more [OR 7.7(2.0–29.6), *p* = 0.003]. Meanwhile, elevated levels of TG (*p* = < 0.001), FBG (*p* = 0.02) and obesity class III (*p* = 0.03) were associated with NAFLD in univariate analysis, but this association was not observed in multivariate analysis (Table 2).

**Table 2:** Predictors of Non-alcoholic fatty liver disease

Variable	Crude Odds Ratio		Adjusted Odds Ratio	
	OR (95%CI)	*P* value	OR (95% CI)	*P* value
Advanced age (> 58 years)	1.5 (0.8–2.8)	0.24	α	α
Female sex	1.2 (0.6–2.4)	0.57	α	α
Staying in urban (ref. rural)	1.7 (0.9–3.1)	0.13	α	α
Overweight	0.5 (0.2–1.3)	0.15	α	α
Obesity class I	0.6 (0.3–1.1)	0.09	2.0 (0.5–2.9)	0.75
Obesity class III	2.7 (1.1–3.4)	0.03	1.1 (0.3–4.0)	0.86
Hypertension	1.7 (0.8–3.8)	0.13	α	α
Diabetes	1.8 (1.0–3.5)	0.06	1.1 (0.5–2.4)	0.19
High systolic blood pressure (≥ 134 mmHg)	1.6 (0.9–3.1)	0.13	α	α
High diastolic blood pressure (≥ 83 mmHg)	1.7 (0.9–3.3)	0.09	1.4 (0.6–3.1)	0.39
High fasting blood glucose (≥ 104 mg/dl)	2.1 (1.1–4.1)	0.02	0.9 (0.4–2.2)	0.81
High triglycerides (≥ 121 mg/dl)	4.5 (2.2–9.0)	< 0.001	0.7 (0.2–2.5)	0.56
Low high-density lipoprotein (≤ 42 mg/dl)	2.1 (1.1–4.0)	0.03	1.8 (0.8–3.9)	0.63
High uric acid (≥ 360 μmol/L)	2.4 (1.3–4.7)	0.008	2.1 (1.1–4.7)	**0.04**
Triglyceride-glucose index ≥8.99	9.4 (4.5–19.3)	< 0.001	7.7 (2.0–29.6)	**0.003**
Triglyceride-glucose index-BMI ≥293.4	3.7 (1.8–7.3)	< 0.001	1.9 (0.8–4.7)	0.16

*OR* odds ratio, *CI* confidence interval, *BMI* body mass index

^α^ Not included in multivariate analysis

**Fig. 1:**
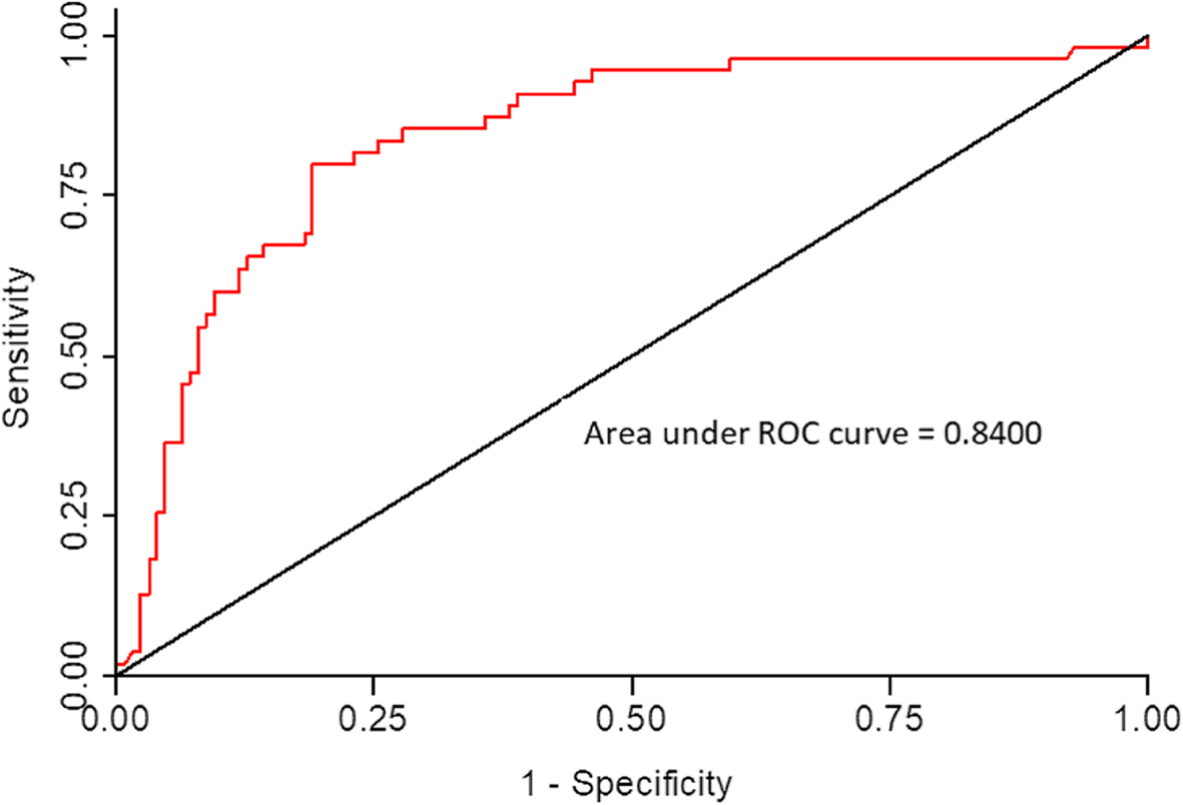
Receiver Operating Characteristics (ROC) Curve and Area Under the Curve for the Test of Goodness of Fit for Multivariate Logistic Model for Predictors of Non-alcoholic fatty liver disease

The Hosmer-Lemeshow goodness-of-fit test did not show an apparent lack of fit, with AUC of 0.8400 (*p* = 0.74) (Fig. 1).

### Correlation between non-alcoholic fatty liver disease with TyG and TyG-BMI

**Fig. 2:**
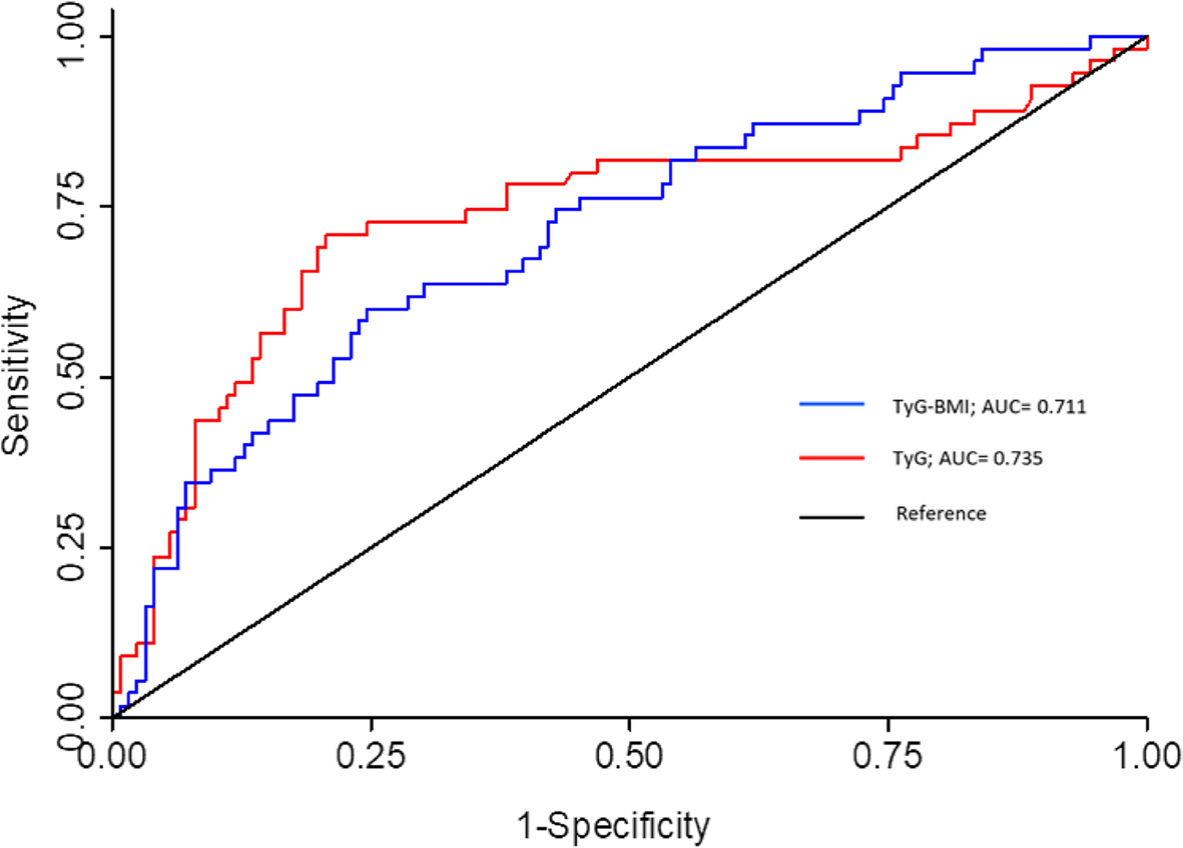
Determination of the TyG and TyG-BMI by receiver operating characteristics (ROC) curve analysis. ROC curve analysis was performed to determine the cut-off points for TyG and TyG-BMI in terms of their accuracy in predicting NAFLD. TyG: Triglyceride-glucose index; TyG-BMI: Triglyceride-glucose index- body mass index; AUC: Area under the curve

The best cut-off points to predict NAFLD were 8.99 for TyG [AUC 0.735; 95% CI (0.66–0.79)], and 312 for TyG-BMI [AUC 0.711; 95% CI 0.64–0.78)] (Fig. 2). By using these cutoffs (8.99 for TyG and 312 for TyG-BMI), the sensitivity of TyG was 70.9% and specificity was 79.3%, and the sensitivity of TyG-BMI was 60% and the specificity was 75.4% for predicting NAFLD compared to ultrasound diagnosis, (Table 3).

**Table 3:** Sensitivity and specificity of the Triglyceride-glucose index and Triglyceride-glucose index-BMI compared to ultrasound in the diagnosis of NAFLD

		Ultrasound diagnosis			Sensitivity	Specificity	PPV	NPV
		NAFLD present	NAFLD absent	Total				
**TyG**	> 8.99	39	26	65	39/55 = 70.9%	100/126 = 79.3%	39/65 = 60.0%	100/116 = 86.2%
	≤8.99	16	100	116				
	Total	55	126	181				
**TyG-BMI**	> 312	33	31	64	33/55 = 60.0%	95/126 = 75.4%	33/64 = 51.6%	95/117 = 81.2%
	≤312	22	95	117				
	Total	55	126	181				

*NAFLD* Non-alcoholic fatty liver disease, *PPV* Positive predictive value, *NPV* Negative predictive value, *TyG* Triglyceride-glucose index, *TyG-BMI* Triglyceride-glucose index-BMI

## Discussion

The current study found a high prevalence (30.4%) of NAFLD in patients with overweight and obesity. There was a strong positive association between hyperuricemia and high TyG and NAFLD risk. We also show a fair correlation between TyG (AUC 0.735) and TyG-BMI (AUC 0.711), and hepatic ultrasound in diagnosing NAFLD, with moderate accuracy.

The prevalence of NAFLD found in this study was lower than in Western countries, with rates reported to be 85% in subjects with grade III obesity and 65% in subjects with grade I-II obesity [15]. In Africa, the magnitude of NAFLD is often underestimated due to limited data. Discrete and few published data are highly variable. A higher prevalence of NAFLD (87%) like that in Western countries has been reported in South Africa in the overweight/obese population [16], while lower prevalence’s of 25 and 12% were reported in obese and overweight participants in Zambia, respectively [17]. This variability can be explained by large differences in the drivers of NAFLD, including dietary composition, lifestyle habits and environmental determinants, due to increased urbanization in western countries compared to low-income countries. NAFLD rates appear to be increasing in proportion to the country’s pace of urbanization. Compared to Western countries where urbanization rates are considered stable, Africa is very dynamic. For example, South Africa is a highly urbanized country compared to Tanzania and Zambia [18], resulting in the highest prevalence of NAFLD compared to the index study. However, our findings within the country showed no variation in the prevalence of NAFLD between individuals living in urban and rural areas. This lack of distinction could be attributed to significant disparities in health outcomes within urban and rural regions, primarily caused by unplanned urban development and unequal economic growth. Consequently, clear definitions between urban and rural areas in Tanzania are absent [19]. According to Levira et al. [20], up to 70% of urban residents in Tanzania reside in impoverished slum areas that are actually poorer than the surrounding rural regions. In our study, we reported generalized data for urban or rural classification without considering the economic status of individuals.

Consistent with the Western studies described above [15], we have showed that not only obesity but also the degree of obesity correlates well with NAFLD. All this, along with the high magnitude of NAFLD that we have reported, serve as a warning to the existence of this silent but serious disease. As this is the first study to report on the extent of NAFLD in the country, it provides the basis and should stimulate further research to obtain definitive national data on the subject.

Compared to previous studies [21], in the index study it has also been shown that NAFLD was diagnosed more frequently among subjects with characteristics of MetS; elevated blood pressures, TG, FBG, decreased HDL, and increased waist circumference. Furthermore, hyperuricemia was found to be a strong independent predictor of NAFLD. A relationship between NAFLD and MetS has been postulated in which MetS is correlated with the promotion of faster progression of NAFLD to NASH, liver fibrosis, and hepatocellular carcinoma, and also patients with MetS characteristics are at high risk of developing NAFLD [2]. Hyperuricemia, although not usually considered an element of MetS, has been correlated with both, increased risk of NAFLD [22] and higher degree of histological liver damage among patients with NAFLD [23]. Indeed, in the index study, almost three-quarters of subjects with advanced liver steatosis were also hyperuricemic. Therefore, it is recommended that subjects with any features of MetS or hyperuricemia are immediately screened for NAFLD and, similarly, those diagnosed with NAFLD, a thorough physical examination and laboratory tests are performed to look underlying metabolic characteristics.

Several studies have reported promising results on the utility of TyG and TyG-BMI in detecting NAFLD. In a large cross-sectional study by Zhang et al. [24], a TyG threshold of 8.52 was found to have a moderate sensitivity of 72.2% and a specificity of 70.5% compared to ultrasound for the detection of NAFLD in the Chinese general population. Another study from Bangladesh showed a high efficacy of TyG (sensitivity 93.5%, specificity 79%; cutoff 8.85) in diagnosing NAFLD [25]. Furthermore, in another study by Ling et al. [26], a close dose-response relationship was found between TyG and NAFLD when TyG was placed in the quartiles. This study showed that each additional unit of TyG was associated with a 2.8-fold increase in the risk of NAFLD. Consistent with these results, TyG shows moderate performance in the diagnosis of NAFLD in the index study (sensitivity 70.9% and specificity 79.3%). However, in Brazil, a study conducted in obese patients undergoing bariatric surgery found a relatively poor performance of NAFLD in predicting NAFLD (sensitivity 67.6 and 65.1%) [27]. Differences between these studies can be explained by the differences in ethnicity and metabolic status among subjects in these studies, which are known to affect TyG effectiveness [26, 28]. These studies did not include black populations.

Due to the strong correlation between BMI and TyG that is known to exist [4, 15], it could be expected that metrics that integrate TyG with BMI would have a greater effect in predicting NAFLD than TyG alone. This notion has been supported by several studies. In their study in Japan, Wang et al. [29] showed a very strong prediction of NAFLD by TyG-BMI. However, this correlation was found to be directly related to reduced age and low BMI. Another study also from Japan, using two TyG-BMI cut-off points to rule out or rule in NAFLD with internal and external validation, showed high efficacy of TyG-BMI to discriminate NAFLD. Up to 70% of all 14,280 participants were able to avoid ultrasound by applying TyG-BMI in this study [30]. Similarly, we observed a strong association between TyG and BMI, but the discriminatory power of the TyG-BMI index in the diagnosis of NAFLD was modest. This finding is plausible, since all of our participants were overweight and obese (median BMI 32 kg / m^2^) and most were elderly (median age 62 years), unlike the Japanese studies above [29, 30] where the median BMI was relatively low (32 kg / m^2^) with younger participants (mean age 43 years). Similar studies recruiting overweight, obese, and elderly people like ours also reported a lower discriminatory rate of TyG-BMI index in the diagnosis of NAFLD [31]. Notably, in these studies, it was suggested that the TyG-BMI index better predicted liver fibrosis than NAFLD, a finding that was beyond our scope. Collectively, these results do not provide sufficiency evidence on the use of TyG and the TyG-BMI index surrogate for hepatic ultrasound in the diagnosis of NAFLD in a black Africans, especially in elderly people with normal weight.

To our knowledge, this is the first study to report the utility of TyG and TyG-BMI index in the diagnosis of NAFLD in black Africans. Therefore, these data provide important background information especially in this population, on the possible use of TyG and the TyG-BMI index as screening tools to guide early intervention. We recognize certain limitations within this study. First, the study was conducted at a single center and was both cross-sectional and retrospective in nature. Further prospective studies involving multiple centers are needed to establish the appropriate thresholds for the indicators used and to determine if TyG and TyG-BMI can accurately predict the occurrence of NAFLD in the future. Second, the limited number of participants may have diminished the predictive effect and diagnostic value of TyG and TyG-BMI. Therefore, further validation through a larger study is necessary before considering these results as a routine clinical option.

## Conclusions

Our study demonstrated that the prevalence of NAFLD is high in overweight and obese populations. The use of TyG and TyG-BMI as a screening tool to identify these individuals for further investigation and timely intervention, need to be further evaluated.

## Data Availability

The datasets used and/or analyzed during the current study are available from the corresponding author on reasonable request.

## Abbreviations

AUC: Area under the curve
BMC: Bugando medical center
BMI: Body mass index
CI: Confidence interval
DM: Diabetes mellitus
FBG: Fasting blood glucose
HDL: High-density lipoprotein
HCC: Hepatocellular carcinoma
IQR: Interquartile range
MetS: Metabolic syndrome
NAFLD: Non-alcoholic fatty liver disease
NASH: Non-alcoholic steatohepatitis
NPV: Negative predictive value
OR: Odd ratio
PPV: Positive predictive value
ROC: Receiver operating characteristic
SBP: Systolic blood pressure
TG: Triglyceride
TyG: Triglyceride-glucose index
